# Active trachoma and associated risk factors among children in Baso Liben District of East Gojjam, Ethiopia

**DOI:** 10.1186/1471-2458-12-1105

**Published:** 2012-12-22

**Authors:** Kassahun Ketema, Moges Tiruneh, Desalegn Woldeyohannes, Dagnachew Muluye

**Affiliations:** 1 Department of Public Health Officer, College of Health Sciences, Debre Markos University, Debre Markos, Ethiopia; 2 School of Biomedical and Laboratory Sciences, College of Medicine and Health Sciences, University of Gondar, Gondar, Ethiopia; 3 Department of Public Health, School of Medicine and Health Sciences, Addis Ababa Science and Technology University, Ethiopia

**Keywords:** Active trachoma, Prevalence, Risk factors, Ethiopia

## Abstract

**Background:**

Trachoma is the leading cause of preventable blindness worldwide. It is common in areas where the people are socio-economically deprived. The aim of this study was to assess active trachoma and associated risk factors among children 1–9 years in East Gojjam.

**Methods:**

Community-based cross-sectional study was conducted in Baso Liben District from February to April 2012. A two-stage random cluster-sampling technique was employed and all children 1–9 years old from each household were clinically assessed for trachoma based on simplified WHO 1983 classification. Data were collected by using semi-structured interview, pre-tested questionnaire and observation. The data were entered and analyzed using SPSS version 16 statistical package.

**Results:**

From a total of 792 children screened for trachoma (of which 50.6% were girls), the overall prevalence of active trachoma was 24.1% consisting of only 17.2% [95% CI: 14.8, 20.1] TF and 6.8% TI. There were variations among children living in low land (29.3%) and in medium land (21.4%). In multivariate analysis, low monthly income (AOR = adjusted odds ratio) 2.98; 95% CI (confidence interval): 1.85-7.85), illiterate family (AOR = 5.18; 95% CI: 2.92-9.17); unclean face (AOR = 18.68; 95% CI: 1.98-175.55); access to water source (AOR = 2.01; 95% CI: 1.27-3.15); less than 20 liters of water use (AOR = 4.88; 95% CI: 1.51-15.78); not using soap for face washing (AOR = 5.84; 95% CI: 1.98-17.19); not using latrine frequently (AOR = 1.75; 95% CI: 0.01-0.42); density of flies (AOR = 3.77; 95% CI: 2.26-6.29); less knowledgeable family (AOR = 3.91; 95% CI: 2.40-6.38) and average monthly income (AOR = 2.98; 95% CI: 1.85-7.85) were found independently associated with trachoma.

**Conclusion:**

Active trachoma is a major public problem among 1–9 years children and significantly associated with a number of risky factors. Improvement in awareness of facial hygiene, environmental conditions, mass antibiotic distribution and health education on trachoma transmission and prevention should be strengthened in the District.

## Background

Trachoma is a contagious eye infection caused by *Chlamydia trachomatis*, serovars A, B, Ba or C. [1]. The transmission of trachoma has been known to occur by fomites, direct contact, and eye-seeking fly *Musca sorbens* which lays its eggs on exposed human feces [2-4]. Communities with endemic trachoma are usually live in dry and dusty area [5] and have been always associated with poverty, poor sanitation and low socio economic status [6].

Trachoma was eliminated from developed countries through improvements of hygiene and sanitation but still a problem of developing countries [7]. It is endemic in more than 50 countries [8]. According to the WHO (2007) report, globally close to 1.3 million people are blind due to trachoma, while about 84 million people suffer from active trachoma (Trachomatous inflammation, follicular (TF) and/or intense (TI)) [8]. Active infection is mostly seen in young children with a peak incidence of around four to six years, while subsequent scarring and blindness is seen in adults [9-11].

The national survey (2007) of Ethiopia showed a prevalence of 40.1% active trachoma among children aged 1–9 years. Amhara region had the highest prevalence of active trachoma among children aged 1–9 years (62.6%) and trichiasis in 15 years and above adults (5.2%) [12]. The high burden of trachoma in the Amhara region calls for collecting a further District-specific data and comprehensive efforts to evaluate the risk factors of trachoma for designing and expanding intervention programs. However, only limited studies were conducted previously, especially on the effect of variation in agro-climates while at present many people in the area are suffering from trachoma in East Gojjam. Among those cases, 64% were from Baso Liben District (unpublished eye clinic records at Debre Markos referral hospital). With this context, this study determined the prevalence of active trachoma among children in this District and identified risky factors that have paramount importance for the improvement of programs aimed at trachoma prevention and control in the area.

## Methods

### Study design

A community based cross-sectional study was carried out in Baso Liben District, East Gojjam, Amhara Regional state, Ethiopia, from February to April, 2012. Baso Liben District is located on latitude and longitude of 10°09^′^N 37°45^′^E coordinates: and 10°09^′^N 37°45^′^E respectively. The area has two agro-climatic divisions: 54% low land and 46% medium land with a land of less than 1500 meters and 1500–2500 meters above sea level, respectively. According to the Central Statistical Agency in 2007, Baso Liben District has a total population of 137,470, of whom 67,594 (49.2%) are men [13]. The number of children aged 1–9 years was about 17,322 [unpublished District health desk information]. The source populations of the study were all children with age of 1–9 years living in Baso Liben District. Five villages of the District were randomly selected as targets of the study. Then children, who met the inclusion criteria from the target villages, were selected as the subjects of the study. Children who had eye injury and/or seriously sick and those who were not at home at the time of study and less than 1 year old were excluded.

### Sampling methods

A total of 792 subjects were enrolled in the study, considering 95% confidence, 5% margin of error, design effect of 2, 62.6% prevalence rate from previous studies [12] and 10% non-response rate. A two-stage random cluster-sampling technique was employed for selecting the study units. In the first stage, five villages were selected using simple random sampling in order to give equal chance for each village. The number of study participants included in each agro-climate and villages were taken proportionally to their population size. In the second stage, households were selected through a random walk method from each village.

### Operational definition

✓ Active trachoma – Trachomatous inflammation, follicles or Trachomatous inflammation intense.

✓ Clean face - a child who did not have an eye discharge or nasal discharge, fly on face at the time of visit.

✓ Head of household - the mother or father, who take care of children most of time.

✓ Knowledge: Knowledge questions were calculated after a number of questions were presented. Correct answers were given score 1 and incorrect answers 0. The sum was computed and those who scored the mean and above were labelled as “knowledgeable” and those who scored below the mean were labelled as “less knowledgeable”.

✓ Low land is an area between 1500 and 2000 meters above sea level.

✓ Medium land is an area between 2001 and 2500 meters above sea level.

✓ Trachoma negative – children that did not have signs of active trachoma.

✓ Trachomatous inflammation, follicles - Five or more follicles of >0.5 mm on the upper tarsal conjunctiva.

### Data collection

Semi-structured questionnaire based interview were used to obtain information on active trachoma. Ten high school students living in the respective villages were trained and interviewed head of the households and observed the children after getting verbal consent from the heads.

Two ophthalmic nurses who had participated in national trachoma survey as eye examiner took part in the examination of the eye. The examination of the eye of each study subjects was done by careful inspection of eye lashes, cornea, limbus, eversion of the upper lid and inspection of the tarsal conjunctiva by the help of magnifying binocular lenses (x2.5) and penlight torches. The guide used for reporting examination results with the simplified trachoma grading scheme was developed by WHO for field work [12,14].

The questionnaires were pre–tested and the necessary corrections were made before the actual data collection. The ophthalmic nurses and data collectors were trained. The principal investigator closely supervised the entire data collection processes. The filled out questionnaires and examination results were collected after checking for consistency and completeness on daily bases. Double entry of 5% data for checking errors was made.

### Data analysis

Data were entered and analyzed using SPSS version 16 statistical package. The analysis part contains descriptive and inferential statistics. Multivariate logistic regression analysis was also conducted to assess the independent effect of each determinant factor after controlling all other factors. P-value < 0.05 was considered as statistically significant association.

### Ethical considerations

This study was approved by Ethical review committee of the University of Gondar, College of Medicine and Health sciences, School of Biomedical and Laboratory Sciences. Permission from District administrative office and villages’ administrators was obtained. The purpose of the study was explained and verbal consents were obtained from all eligible children’s head of households who took part in the study. All children with active trachoma were treated with two tube of tetracycline eye ointment.

## Results

### Socio-demographic characteristics

A total of 792 children aged l-9 years from 365 households of five villages were systematically selected. The mean ± standard deviation (SD) age of children was 4.93 ± 2.35 years. Of all, 401 (50.6%) were girls and most of the children 540 (68.2%) were preschool age whereas 203(25.6%) were attending school. Regarding their agro-climatic residential areas there were 273 (34.5%) and 519 (65.5%) children from low and medium land villages, respectively. The majority of head of households 252 (69.9%) were females. Almost all study participants at household level 362 (99.6%) were Amhara and 362 (99.6%) of them were Orthodox Christians. Three hundred eleven (85.2%) of respondents were farmers. Two hundred nineteen (60%) of heads of households were illiterate while 146 (40%) had attended primary school and above. The average monthly income of most households 443 (55.9%) was less than or equal to 600 Ethiopian birr (ETB). The average family size among the population was 5.43 (Table 1).

**Table 1:** Socio-demographic characteristics of heads of household in Baso Liben District from February to April, 2012

**Characteristics**	**Frequency (N = 365)**	**Percentage (%)**
**Sex of head of household**		
Male	110	30.1
Female	255	69.9
**Age in years**		
18-29	170	46.6
30–44	134	36.7
45–59	41	11.2
60+	20	5.5
**Occupation**		
Farmer	311	85.2
Merchant	33	9.1
Others	21	5.7
**Education status**		
Illiterate	219	60
Primary and above	146	40
**Average Monthly income**		
<600	443	55.9
601–1200	239	30.2
1201–2000	75	9.5
>2000	35	4.4

### Active trachoma among children of 1–9 years old

**Figure 1:**
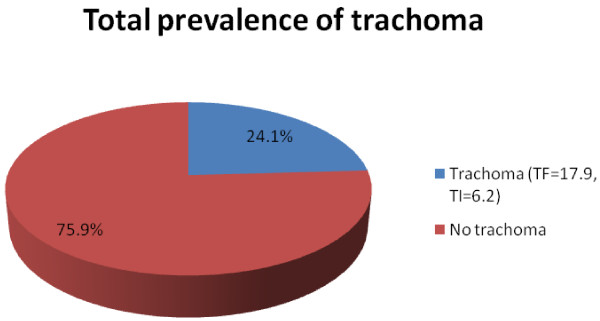
Prevalence of active trachoma in children 1–9 years in Baso Liben district from February to April, 2012.

**Figure 2:**
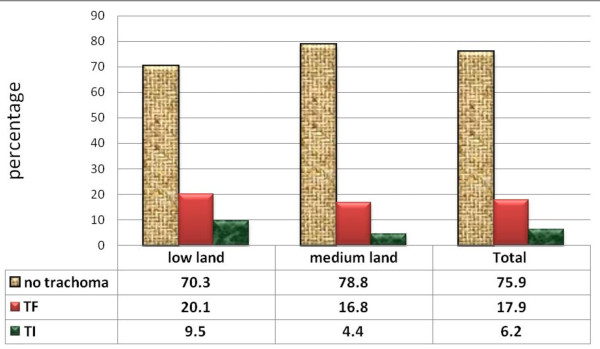
Prevalence of active trachoma in study subjects in low land and medium land in Baso Liben District from February to April, 2012.

The overall prevalence of active trachoma was 191 (24.1%). Of these, 137 (17.2%) were TF cases while 54 (6.9%) were TI cases (Figure 1). Among all cases of TI, 29 (53.1%) were girls. The proportion across the ages increases reaching peak of 34 (27.9%) at the age 4 years and slowly decreases as age increases. There were agro-climatic residence area variations in the active trachoma among children which was 80 (29.3%) in low land and 111 (21.4%) in the medium. Moreover, TF and TI were more common [(20.1% versus 9.5%) in low land relative to (16.8% versus 4.4%) in medium land respectively] (Figure 2). Number of illiterate household heads in low land were 208 (76.2%) and 267 (51.4%) in medium land; 53.8% households have no latrine in low land but only 27.4% in medium land; more flies on face of children 9.5% were in low land and 3.1% in medium land. Most of household heads 192 (70.5%) of low land have less knowledge on trachoma while 323 (62.2%) in medium land. Number of illiterate household heads, absence of latrine and presence of more flies on the face of children were found statistically significant for agro-climatic area variations.

The distribution of active trachoma was different in different age groups. TF was very high at the age one and four years. Among age groups, 4–6 years old children 84 (10.6%) were more infected than 1–3 years old (7.3%) and 7–9 years old (6.2%) children.

### Factors associated with active trachoma

In multivariate analysis; proxy indicators of low socioeconomic status, educational status of households, access to water source, amount of water consumption per capita, frequency of latrine usage, usage of soap, unclean face, number of fly on face of a child and awareness status of households on trachoma were found independently associated with active trachoma.

Children from illiterate heads of households 170 (35.8%) were 5 times (AOR = 5.19; 95% CI 2.93-9.18) more likely to have active trachoma than those from educated ones 21 (6.6%). The prevalence of trachoma infection decreases from 148 (18.7%) to 0 (0%) as average monthly income of their family increases from less than or equal to 600 to greater than 2000 ETB (AOR = 2.98; 95% CI 1.86-7.86). The prevalence of active trachoma among girls (24.7%) and boys (23.5%) was almost similar. More children from households having water access in more than 30 minutes distance 111 (35.7%) were found with active trachoma than those who could access water within 30 minutes or less foot travel distance 80 (16.6%) (AOR = 2.008; 95% CI 1.28-3.16). The prevalence of active trachoma was also high in children from parents with average daily water consumption of 2–4 liters/capita, 313 (39.5%) than those having more than 8 liters/capita 59 (7.4%) (AOR = 5.45; 95% CI 1.85-16.02). The common sources of water for domestic consumption in the study area were river 447 (56.4%), tap water 201 (25.4%) and other sources 144 (18.2%). Active trachoma was more prevalent in children who did not frequently wash their face 7 (31.8%) than those who washed their face more than once per day 22 (11.1%) (AOR = 5.84; 95% CI 1.98-17.19). Children with unclean face were about 18 times more likely to have active trachoma than those with clean faces (Table 2).

**Table 2:** Association of active trachoma and potential risk factors among children 1–9 years in Baso Liben District from February to April, 2012

**Characteristics**	**Trachoma status**		**Crude OR (95%CI)**	**Adjusted OR (95%CI)**
	**Total examined N (%)**	**Positive N (%)**		
**Agro-climate**				
Low land	273(34.5)	80(29.3)	1.52(1.09-2.12)*	1.12(0.58-2.16)
Medium land	519(65.5)	111(21.4)	1	
**Educational status of head of household**				
Illiterate	219(59.2)	78(35.8)	7.85(4.85-12.70)***	5.18(2.93-9.17)***
primary& above	146(40.8)	10(6.6)	1	
**Average monthly income**				
<600	443(55.9)	148(18.7)	4.60(2.81- 7.53)***	2.98(1.85-7.85)***
601–1200	239(30.2)	41(5.2)	2.37(1.37- 4.09)	3.89(1.87-8.09)
1201–2000	75(9.5)	2(0.3)	1.01(.53- 1.93)	1.29(0.56-3.81)
>2000	35(4.4)	0(0)	1	
**Access to water source**				
≤ 30 minutes walk away	729(92)	209(28.7)	1	
> 30 minutes walk away	63(8)	8(12.7)	2.78(1.99-3.88)***	2.01(1.27-3.15)**
**Daily average Water consumption**				
< 20 liters per head	160(20.2)	41(25.60	2.55(1.07-6.08)***	4.88(1.51-15.78)**
20–40 liters per head	313(39.5)	100(31.9)	3.48(1.53-7.55)	5.45(1.85-16.01)
40–60 liters per head	152(19.2)	18(11.8)	0.99(0.39-0.2.52)	1.46(.451-4.77)
60–80 liters per head	108(13.6)	25(23.1)	2.23(0.90-5.54)	2.37(.72-7.74)
>80 l per head	59(7.5)	7(11.9)	1	
**Frequency of larine usage**				
Always	387(76.7)	39(10.1)	1	
Sometimes	117(23.3)	53(45.3)	1.58(0.14-0.32)***	1.75(0.12-0.42)**
**Usage of soap**				
Yes	250(31.5)	22(8.8)	1	
No	542(68.5)	169(31.2)	4.69(2.92-7.54)***	5.84(1.98-17.19)**
**Clean face of child**				
Clean face	46(5.8)	1(2.2)	1	
Unclean face	746(94.2)	190(25.5)	15.37(2.10-11.32)**	18.68(1.98-175.55)*
**Number of fly on face of child**				
0–3	546(69.9)	73(13.4)	1	
4–7	204(25.7)	94(46.1)	5.53(3.82-8.01)***	3.77(2.26-6.29)**
>7	42(5.3)	24(57.5)	8.63(4.47-16.69)	4.47(1.86-10.70)
**Knowledge of heads of household**				
Knowledgeable	188(51.5)	30(16.6)	1	
Less knowledge	177(48.5)	57(32.1)	2.37(1.69-3.32)***	3.91(2.40-6.38)***

***Significant at p-value ≤0.001, ** significant at p-value ≤ 0.01, * significant at p-value ≤0.05.

Most of the children from households who claimed using latrine always had less chance of developing infection compared to those using latrine occasionally and it was statistically significant. The prevalence of active trachoma among children from households with less awareness about trachoma were about four times more likely to develop trachoma compared to those from knowledgeable families (AOR = 3.92; 95% CI 2.41-6.38) (Table 2).

## Discussion

The findings of this study showed overall prevalence of 24.1% active trachoma (range, 21.4–29.3%). Even if it is decreasing in figure compared to Amhara regional prevalence, 62.2% in 2007 [15], the finding of the study confirmed that trachoma is still a disease of public health interest. This result was almost in agreement with studies in other countries like in Malawi, 25.1% (16). The possible explanation could be increment of mass antibiotic distribution coverage with weak integration of health promotion on primary eye care, health information on personal and environmental hygiene of the Districts. The prevalence was higher than the study in Ghana (16.1%) [16]. This might be due to differences in endemicity and period of study.

The prevalence of active trachoma was higher in lowland than medium land agro-climatic residence area. The potential risk factors related to agro-climatic area differences were illiterate household heads, absence of latrine, more flies on face of children. Children from illiterate household heads were 5 times more likely to have active trachoma than from literate household heads. This result was consistent with a study conducted in Tigray region, Ethiopia [17]. Similar finding was reported from Tanzania [18]. These might be due to the effect of literacy of the father/mother which is especially important because she/he is responsible for the caretaking of the children. An educated mother may be more aware of the benefits of hygiene practices to the health of her children compared to an uneducated mother.

Children from households getting less monthly income were more likely to have trachoma than those from households getting more income. A similar outcome was reported in Ethiopia [6]. The possible explanation could be due to effects of poverty on health care, lack of hygiene, high chance of sharing tools, immunity status, and lack of information.

Those families who walk more than 30 minutes distance from water source were two times more likely to have trachoma than those on less than 30 minutes walk from water bodies. This finding was in line with investigations of Tanzania and Ethiopia [19-21].

The study has also revealed more association between active trachoma and frequency of latrine use than presence of latrine. This result is similar with previous report in Ankober, Ethiopia [21]. This could be due to inaccessibility to latrine facilities and frequent exposed of human feces which are risk factors for the presence of high number of fly-eyes that leads to high chance of transmission of trachoma.

In the present study, there was significant association between active trachoma and unclean face. This finding is similar to studies that identify the presence of ocular and nasal discharge as risk factors for the presence of fly on eyes and active trachoma in Ethiopia and Tanzania [18,22]. The presence of more flies on face was associated with more chance of having active trachoma. This finding is consistent with different investigations in Brazil; Nigeria and Ethiopia [23-25]. The possible explanation could be the role of eye seeking fly in trachoma transmission which is still remained common and high.

Children with less knowledgeable household heads about trachoma had about four times more likely to have infection than children from knowledgeable household heads. This finding was in line with investigations in Ethiopia which also reported overall reduction of trachoma ranges from 4% to 12% after provision of health education for community [26,27]. Similar result was reported by the study conducted in Tanzania on comparing effect of antibiotic treatment alone versus antibiotic treatment combined with an intensive health education programme about facial cleanliness [28]. The possible explanation for the differences could be less access to information, education and communication media on trachoma prevention, community based health education by trained health workers or volunteers and eye care units in the District.

The limitation of the study were the estimation of economic status, distance of water source and average monthly income of study households. It was merely based on respondents’ reply to the interviewer questions, which may be uncertain. Due to lack of sufficient resources the positive result of trachoma status could not be confirmed by advanced laboratory tests so that differential diagnosis may exaggerate the result. Moreover, the small sample size used might have an effect on the real magnitude of active trachoma.

## Conclusion

The prevalence of active trachoma among children 1–9 years old in Baso Liben District exceeds the WHO guidelines for the intervention with mass antibiotic distribution which warrants the trachoma SAFE control strategy to be strengthened in the District. A number of risky factors were significantly associated with prevalence of active trachoma. These include low socioeconomic and/or educational status of heads of households, distance of water source from home, average water consumption, frequency of usage latrine and soap among others. Unclean face, density of fly on face of a child and awareness of head of households about trachoma were also determinant factors for the occurrence of active trachoma. Access to sanitary water and frequently using latrines are essential factors of the ‘E’ component of the SAFE strategy. Moreover, there was higher prevalence of active trachoma in low land than medium land villages that needs special attention in preventive programs.

## Competing interests

The authors declare that they have no competing interests.

## Authors’ contributions

KK: conception and initiation of the study, design, implementation, analysis and writing. MT: design, implementation of the study and co-writing. DW: design, implementation and co-writing. DM: design, implementation, analysis and co-writing. All authors read and approved the final manuscript.

## Pre-publication history

The pre-publication history for this paper can be accessed here:

http://www.biomedcentral.com/1471-2458/12/1105/prepub
